# The Meditative Mind: A Comprehensive Meta-Analysis of MRI Studies

**DOI:** 10.1155/2015/419808

**Published:** 2015-06-04

**Authors:** Maddalena Boccia, Laura Piccardi, Paola Guariglia

**Affiliations:** ^1^Department of Psychology, “Sapienza” University of Rome, Via dei Marsi 78, 00185 Rome, Italy; ^2^Neuropsychology Unit, IRCCS Fondazione Santa Lucia, Via Ardeatina 306, 00179 Rome, Italy; ^3^Department of Life, Health and Environmental Sciences, L' Aquila University, P.le S.Tommasi 1, 67100 Coppito, Italy; ^4^Department of Human Science and Society, University of Enna “Kore,” Cittadella Universitaria, 94100 Enna, Italy

## Abstract

Over the past decade mind and body practices, such as yoga and meditation, have raised interest in different scientific fields; in particular, the physiological mechanisms underlying the beneficial effects observed in meditators have been investigated. Neuroimaging studies have studied the effects of meditation on brain structure and function and findings have helped clarify the biological underpinnings of the positive effects of meditation practice and the possible integration of this technique in standard therapy. The large amount of data collected thus far allows drawing some conclusions about the neural effects of meditation practice. In the present study we used activation likelihood estimation (ALE) analysis to make a coordinate-based meta-analysis of neuroimaging data on the effects of meditation on brain structure and function. Results indicate that meditation leads to activation in brain areas involved in processing self-relevant information, self-regulation, focused problem-solving, adaptive behavior, and interoception. Results also show that meditation practice induces functional and structural brain modifications in expert meditators, especially in areas involved in self-referential processes such as self-awareness and self-regulation. These results demonstrate that a biological substrate underlies the positive pervasive effect of meditation practice and suggest that meditation techniques could be adopted in clinical populations and to prevent disease.

## 1. Introduction

Mind and body practices such as yoga, meditation, progressive relaxation, or guided imagery use mental and physical abilities to improve health and well-being. Over the past decade these practices have received increasing attention in different fields of study in which the physiological mechanisms underlying the beneficial effects observed in trained individuals have been investigated. Increased knowledge about the physiological effects of mind and body practices makes it possible to explore their therapeutic potential, identify adverse effects, and safely integrate these techniques into standard therapeutic approach.

Meditation is a complex process aimed at self-regulating the body and mind and is often associated with psychological and neurophysiological modifications [1]. Meditation practices can be oriented toward the concentration of attention on a particular external, corporal, or mental object, while ignoring all irrelevant stimuli (focused attention meditation), or toward techniques that try to enlarge the attentional focus to all incoming sensations, emotions, and thoughts from moment to moment without focusing on any of them (open monitoring meditation) [2]. In any case, most meditation approaches use both types of practices complementarily [3, 4].

Meditation practice has been found to promote well-being by fostering cognitive and emotional processes [5, 6]. Specifically, it has been found to improve working memory and attentional processes [7–9] as well as perceptual abilities [10]. It has also been found to promote prosocial behavior [11] and emotional regulation [12]. The potential contribution of meditation to cognitive and emotional processes can be appreciated in the context of the model proposed by Lutz and colleagues [13, 14]. These authors posited that meditation practice induces enhancement of at least four different abilities: sustained attention, monitoring faculty (to detect mind wandering), the ability to disengage from a distracting object without further involvement (attentional switching), and the ability to redirect focus to the chosen object (selective attention). A recent systematic review by Chiesa and colleagues [15] allowed drawing some important conclusions about the positive effect of meditation on cognitive functions. Executive functions, attention, and memory were the main targets of meditation practice. In particular, as compared to the control group, meditators showed improved sustained attention [16], conflict monitoring [7], and reduced attentional blink [17]. Meditators also performed better than controls in the classical working memory paradigms [16, 18]. Concerning memory, significant improvement was found in meta-awareness [19] and in specific autobiographical memories [20] after meditation training. Ortner and colleagues [21] also found that meditation groups showed reduced interference from unpleasant pictures, suggesting that meditation also has a positive effect in decreasing emotional interference during performance of a cognitive task.

Interestingly, the current literature suggests that meditation has a potential effect on age-related cognitive decline [22, 23], probably due to the regulation of glucocorticosteroids, inflammation, and serotonin metabolism [23]. Furthermore, it has been hypothesized [24] that the stress reduction promoted by meditation contrasts hippocampal vulnerability to neurotoxicity [25] and leads to increased hippocampal grey matter volume due to neuron preservation and/or neurogenesis. Meditation has also been found to reduce a number of psychological and physical symptoms in clinical populations [26, 27]. King and coworkers [28] found that mindfulness-based cognitive therapy was an acceptable brief intervention therapy for combatting PTSD: indeed, it reduced avoidance symptoms and PTSD cognitions. There is also evidence that, compared to standard care, mindfulness-based cognitive therapy almost halves the risk of relapse in people who are currently well but who have experienced at least three prior episodes of depression [29, 30] and is comparable to antidepressant medication in reducing risk of relapse [31].

The effects of meditation on brain structure and function have received increasing attention in neuroimaging studies (MRI, fMRI, and PET) and the number of published studies is steadily growing [32]. Specifically, the findings of neuroimaging investigations have allowed linking the positive effects of meditation to specific brain modifications. Neuroimaging studies of brain modification can be roughly divided into those investigating (1) neurofunctional correlates of meditation, (2) neurofunctional modifications after meditation training, and (3) structural brain modifications in expert meditators.

*Functional studies on the brain correlates of meditation* have assessed neural activation during meditation by requiring participants to undergo fMRI scans during meditation tasks. These studies have reported increased activation in areas associated with attention, mind wandering, retrieval of episodic memories, and emotional processing during meditation [33]. Specifically, increased activation in the prefrontal cortex [34], parietal areas [35], middle cingulate cortex, and hippocampal and parahippocampal formations [36] has been reported.

Studies of* functional brain modifications after meditation training *have focused on functional and the metabolic changes after meditation training and/or in expert meditators compared with control participants. These studies adopted different paradigms (Table 1): the affective Stroop task [37], pain-related tasks [38–41], attentional paradigms [42–44], emotional provocation [45], and meditation tasks [36, 46, 47]. The results of these studies are very intriguing because they shed more light on the possible link between neurofunctional changes and the positive effect of meditation on different aspects of cognitive and emotional processes, such as perceptual and attentional processes [7–10] as well as social behavior [11] and emotional regulation [12].

The studies that investigated* structural brain modifications* in expert meditators (Table 2) focused on brain structural changes after meditation training and/or in expert meditators compared with control participants; they primarily assessed grey matter changes with whole-brain voxel-based morphometry or cortical thickness mapping of MRI data [24, 48–54]. These studies principally found that, compared with control participants, expert meditators showed increased grey matter volume at the level of the posterior cingulate cortex, temporoparietal junction, angular gyrus, orbitofrontal cortex, hippocampus, and subiculum in the medial temporal lobe and the brainstem.

Previous neuroimaging studies on the effects of meditation on brain structure and function adopted different meditation techniques and recruited participants with different meditation training. For example, some studies recruited Buddhist practitioners [44] and others recruited participants with experience in SOHAM meditation [47] or ACEM meditation [33]. Several studies reported that different meditation techniques require different cognitive processes and thus produce different neural effects [55, 56]. But, despite differences in meditation techniques and underlying cognitive processes, it has been proposed that all meditation techniques share a central process that supports their common goal, that is, inducing relaxation, regulating attention, and developing an attitude of detachment from one's own thoughts [57]. Evidence from a recent meta-analysis of ten neuroimaging studies [57] seems to suggest that the caudate body, entorhinal cortex, and medial prefrontal cortex have a central role in supporting the general aspects of meditation effects.

The large amount of data collected over the past decade allows drawing some definite conclusions about the neural effects of meditation practice and allows discussing the positive effects of meditation practice from a biological point of view.

The main aim of the present study was to draw some definite conclusions about the neural network activated during meditation tasks and to explore functional (fMRI) and structural (sMRI) changes in expert meditators. To pursue this aim we adopted a meta-analytic approach based on activation likelihood estimation (ALE) analysis, which allows performing coordinate-based meta-analyses of neuroimaging data [58].

## 2. Method

### 2.1. Inclusion Criteria for Papers

The database search on PubMed was performed using the following string: (((((((MRI) AND meditation) NOT Alzheimer's) NOT Parkinson's) NOT EEG) NOT MEG) NOT mild cognitive impairment). A total of 93 papers emerged. From this collection, we selected only papers that (1) included whole-brain analysis performed using magnetic resonance imaging (MRI), (2) provided coordinates of activation foci either in Montreal Neurological Institute (MNI) or in Talairach reference space, (3) studied young and healthy participants, (4) reported activation from group studies, (5) included meditators or required participants to perform a meditation task, and (6) used no pharmacological manipulation. We selected 57 papers: 42 reported fMRI studies and 15, sMRI studies. Out of the 42 fMRI studies, 5 were excluded because they did not provide coordinates of activation foci; out of the 15 papers on sMRI studies, 6 papers were excluded for the same reason and one paper was excluded because it did not include expert meditators.

In line with the aims of the present meta-analysis, individual experimental studies from selected papers were divided according to three main axes: papers reporting (a) functional magnetic resonance imaging studies (fMRI) during meditation training, (b) functional magnetic resonance imaging studies (fMRI) that studied neural modifications after meditation training, and (c) structural MRI studies (sMRI). Note that the fMRI studies on neural modifications after meditation (see Table 1) included those that adopted different paradigms. These studies also reported the results of comparisons between pre- and posttreatment or results of comparisons between expert meditators and naïve participants. A meta-analytic approach, which models the probability distributions centered at the coordinates of each activation focus, allows obtaining a general picture of functional neural modifications in meditators.

We included 37 individual fMRI experimental studies on functional activations during meditation tasks (642 participants), 63 fMRI experimental studies (see Table 1 for more details) on functional changes ascribable to meditation (1,652 participants including both meditators and controls), and 10 experimental sMRI studies (Table 2) on structural changes ascribable to meditation (581 participants).

### 2.2. Activation Likelihood Estimation (ALE)

Activation likelihood estimation (ALE) analyzes the probability that a voxel will contain at least one of the activation foci; it is calculated at each voxel and results in a thresholded ALE map. In other words, ALE assesses the overlap between foci by modeling the probability distributions centered at the coordinates of each one [58].

Our first aim was to provide a general picture of areas activated during meditation tasks. Thus, we carried out an ALE analysis of fMRI studies on functional activations during meditation tasks. Then, we performed two ALE analyses to determine whether meditation produces consistent modifications in brain structure and function. In the first analysis we included sMRI studies, and in the second analysis we included fMRI studies on neural modifications after meditation training.

The ALE meta-analysis was performed using GingerALE 2.1.1 (http://brainmap.org/) with MNI coordinates (Talairach coordinates were automatically converted into MNI coordinates by GingerALE.). Following Eickhoff et al.'s modified procedure [58], the ALE values of each voxel in the brain were computed and a test was performed to determine the null distribution of the ALE statistic of each voxel. The FWHM value was automatically computed because this parameter is empirically determined [58].

For the fMRI studies, the thresholded ALE map was computed using *P* values from the previous step and a false discovery rate (FDR) at the 0.05 level of significance (Tom Nichol's FDR algorithm). Moreover, a minimum cluster size of 200 mm^3^ was chosen. A cluster analysis was performed on the thresholded map.

For the sMRI studies, the thresholded ALE map was computed using *P* values from the previous step and a cluster level correction at the 0.05 level of significance, with a minimum cluster size of 200 mm^3^. A cluster analysis was performed on the thresholded map.

The ALE results were registered on an MNI-normalized template (http://brainmap.org/) using Mricro (http://www.mccauslandcenter.sc.edu/mricro/index.html).

## 3. Results 

### 3.1. Brain Areas Activated during Meditation Tasks

**Figure 1:**
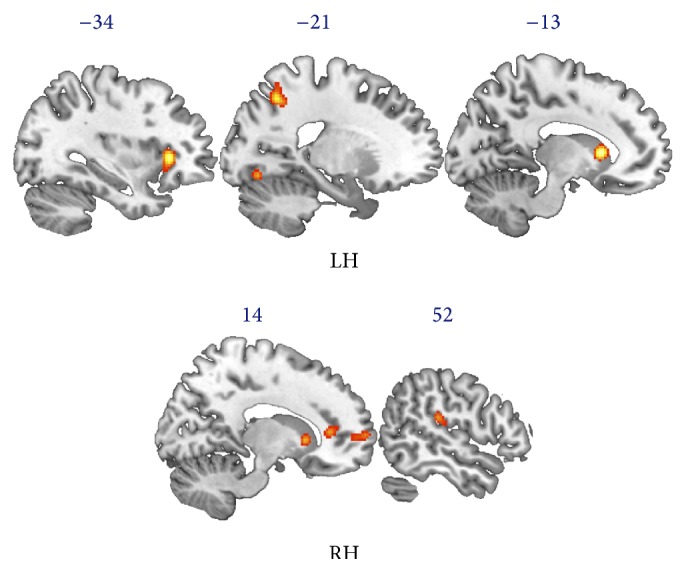
Results of ALE analysis on fMRI studies of meditation. The ALE map shows brain areas activated during meditation, encompassing bilaterally the caudate nuclei and insula, precuneus, middle and superior temporal gyrus, and precentral gyrus in the left hemisphere (LH) and the anterior cingulate cortex, superior frontal gyrus, parahippocampal gyrus, inferior parietal lobule, and middle occipital gyrus in the right hemisphere (RH).

ALE meta-analysis of fMRI studies carried out during meditation revealed a network of areas spanning from the occipital to the frontal lobes that was more highly activated during the meditation condition than the control condition. This network included the caudate nuclei and insula bilaterally, the precuneus, middle and superior temporal gyrus, and precentral gyrus in the left hemisphere, and the anterior cingulate cortex, superior frontal gyrus, parahippocampal gyrus, inferior parietal lobule (angular gyrus), and middle occipital gyrus in the right hemisphere. We also found that left posterior cerebellum, specifically the declive, was more highly activated during meditation than the control condition (Figure 1).

### 3.2. Functional Modifications in Meditators

**Figure 2:**
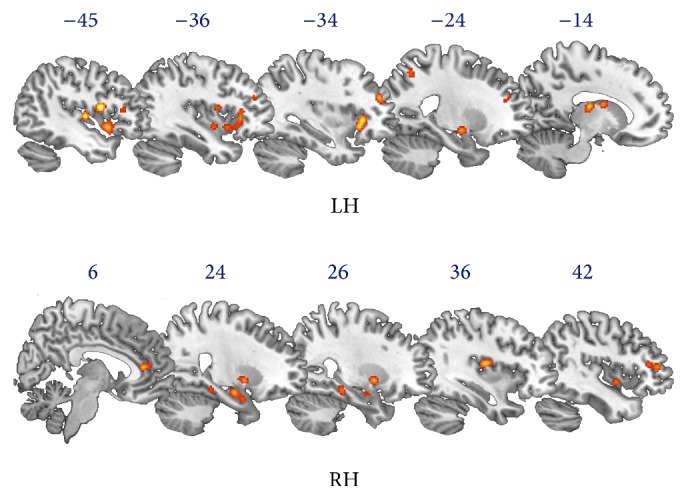
Results of ALE analysis on functional modifications in meditators. The ALE map shows brain areas that are more highly activated in meditators than controls. This network includes bilaterally the middle frontal gyrus, precentral gyrus, anterior cingulate cortex, insula, and claustrum. In the left hemisphere (LH) we found activation of the inferior frontal gyrus, precuneus, caudate nucleus, and thalamus, and in the right hemisphere (RH) we found activation in the medial frontal gyrus, parahippocampal gyrus, middle occipital gyrus, inferior parietal lobule, and lentiform nucleus.

We found that meditation practice (see Table 1) was associated with increased functional activation in a wide network of areas including the bilateral middle frontal gyrus, precentral gyrus, anterior cingulate cortex, insula, and claustrum. In the left hemisphere we also found increased activation at the level of the inferior frontal gyrus, precuneus, caudate nucleus, and thalamus; and in the right hemisphere we found increased activation in the medial frontal gyrus, parahippocampal gyrus, middle occipital gyrus, inferior parietal lobule, and lentiform nucleus (Figure 2).

### 3.3. Structural Modifications in Meditators

**Figure 3:**
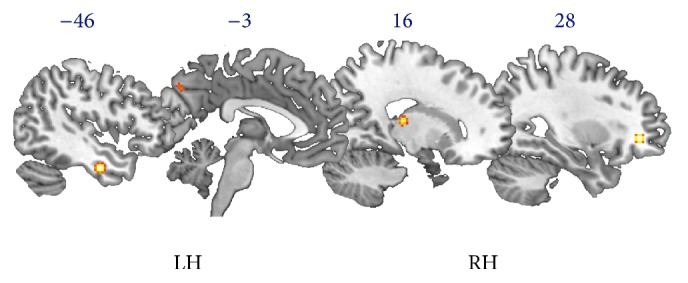
Results of the ALE analysis of structural modifications in meditators. The ALE map shows increased grey matter volume in meditators in the right hemisphere (RH) at the level of the anterior cingulate cortex and thalamus and in the left hemisphere (LH) at the level of the middle and medial frontal gyrus, precuneus, and fusiform gyrus.

We found that meditation practice was associated with increased grey matter volume in the frontal lobe, at the level of the right anterior cingulate cortex and left middle and medial frontal gyrus. We also found increased grey matter volume in meditators at the level of the left precuneus and fusiform gyrus and the right thalamus (Figure 3).

## 4. Discussion

The main aim of the present study was to identify the neural network activated during meditation and to explore structural and functional brain modifications in expert meditators. We also aimed to explore the relationship between meditation practice and the neural mechanisms that allow maintaining the positive effects of meditation training. For this purpose we adopted ALE analysis, a technique used widely in coordinate-based meta-analyses of neuroimaging data [58]. The results of this study shed light on the neural underpinnings of the positive effects of meditation practice and suggest the existence of a neural network responsible for these effects in meditators' everyday life.

The first question we tried to answer was* which* brain areas were activated during mediation. We used ALE analysis to identify the neural networks activated during meditation tasks and carried out the coordinate-based meta-analysis on experimental studies that required participants to meditate during the fMRI scan, regardless of their previous experience. We found that a set of brain areas spanning from the occipital to the frontal lobes was more highly activated during the meditation condition than during the control condition. This network included areas involved in processing self-relevant information, such as the precuneus [59], in processing self-regulation, focused problem-solving, and adaptive behavior, such as the anterior cingulate cortex [60], in interoception and in monitoring internal body states, such as the insula [61], in reorienting attention, such as the angular gyrus [62], and in processing the “experiential enactive self,” such as the premotor cortex and superior frontal gyrus [63]. It is not surprising that meditation induces higher activation in all of these areas, because the mental state during meditation is mainly characterized by full attention to internal and external experiences as they occur in the present moment [15].

As previously described, meditation practice has been found to promote well-being by fostering cognitive and emotional functioning [6]. Indeed, the positive effects achieved during the training sessions were generalized to everyday life, enhancing both cognitive (i.e., memory, attention, problem-solving, and executive functions) and emotional (i.e., prosocial behavior) functioning in expert meditators. Using the ALE method, we tried to address the question about the brain underpinnings of pervasive positive effects of meditation in expert meditators' daily lives. We carried out an ALE analysis that included studies which compared activations in expert meditators and control participants in a wide range of cognitive and emotional domains (Table 1). Results of the ALE analysis showed that meditators, as compared with controls, showed greater activation in a wide network of areas encompassing bilaterally the frontal, parietal, and temporal regions. In addition to areas also activated during meditation (i.e., the middle occipital gyrus, inferior parietal lobule, precuneus, anterior cingulate cortex, precentral gyrus, insula, and caudate nuclei), this network of areas also included thebilateral middle frontal gyrus, inferior frontal gyrus, and thalamus in the left hemisphere and the medial frontal gyrus and lentiform nucleus in the right hemisphere. The network of areas we found more highly activated in expert meditators than in nonmeditators has recently been hypothesized to be part of the enactive experiential self network, which integrates efferent and reafferent processes concerning exteroception, proprioception, kinesthesia, and interoception [63]. Furthermore, it was previously thought that these areas were involved in self-referential processes [64–66], perspective taking [67], cognitive distancing [68–71], and sustained attention [72]. In fact, they were found to be more highly activated in Buddhist meditators [1]. Expert meditators also showed higher activations in the parahippocampal cortex, which has repeatedly been found to be involved in memory formation and retrieval [73, 74] as well as in high-level perception, especially in perceiving complex and ambiguous visual stimuli [75, 76]. The higher activation we found in expert meditators may account at least in part for enhanced attention, memory, and perceptual abilities reported in previous studies [15].

Results of the ALE analysis of sMRI studies showed increased grey matter volume in meditators compared to control groups in the right anterior cingulate cortex, left middle and medial frontal gyrus, left precuneus and fusiform gyrus, and right thalamus. It could be that the increased grey matter volume in the anterior cingulate cortex of meditators accounts for the improvement of specific abilities such as self-regulation, self-control, focused problem-solving, and adaptive behavioral responses under changing conditions [60], which are strictly associated with the functioning of the anterior cingulate cortex. Furthermore, the anterior cingulate cortex has recently been proposed to mediate the positive effects of meditation on prosocial behavior [63]. Nevertheless, it is difficult to state whether this difference as well as many other aspects of cognitive functioning is due to meditation practice or to previous individual predisposition. Studies comparing individuals before and after meditation training may help to clarify this point. Hölzel and colleagues [48] found increased grey matter concentration from pre- to post-MBSR training at the level of the temporoparietal junction, cerebellum, and posterior cingulate cortex. Furthermore, Kurth and colleagues [50] found a shifting in brain asymmetry at the level of the precuneus that was significantly correlated with number of years of practice. These results, taken together with results of the present ALE meta-analysis, which also found structural change in precuneus volume in meditators compared to controls, suggest that while structural differences at the level of the anterior cingulate cortex dispose to meditation, structural changes after meditation are strongly associated with changes in the posterior cingulate cortex and precuneus. The precuneus, which is located in the posteromedial portion of the parietal lobe, was recently found to be involved in a wide range of highly integrated tasks such as visuospatial imagery, episodic memory retrieval, and self-processing operations [59]. It shows widespread connectivity patterns with cortical and subcortical brain regions, such as the prefrontal cortex, anterior cingulate cortex, claustrum, caudate nucleus, and putamen [59]. The wide range of precuneus connections could account for its involvement in many high-level cognitive tasks. Specifically, involvement of the precuneus in self-referential processing could explain why it is so important in meditation practice. The precuneus was found to be involved in self-relevant information processing when self-relevant traits were compared with self-irrelevant traits [77]. It was also found to be involved during the performance of goal-directed actions when compared with passive stimulus viewing [78], the conscious resting state [79, 80], and the enhanced consciousness state of yoga meditation [55]. All of this evidence converges to suggest that the precuneus has a pivotal role in sustaining the positive effects of meditation practice especially because of its involvement in gathering self-relevant information and in representing the self and the external world [59].

Regarding the differences among meditation techniques, as reported above, meditation practices can be grossly divided into two different approaches: focused attention meditation and open monitoring meditation. Anyway, most meditation approaches use both types of practices complementarily [3, 4] and it has been proposed that all meditation techniques share a central process that supports their common goal, that is, inducing relaxation, regulating attention, and developing an attitude of detachment from one's own thoughts [57]. Our results strongly support the existence of a dedicate brain network that supports the general aspects of meditation effects. Actually, other than confirming the role of the caudate body, entorhinal cortex, and medial prefrontal cortex [57], the present study, using a large sample of experimental studies, sheds some light on other sets of brain areas which may be essential in supporting the general aspects of meditation effects.

## 5. Conclusions

Overall, results of the present ALE analysis suggest that meditation practice induces functional and structural brain modifications, especially in areas involved in self-referential processes, including self-awareness and self-regulation [63], as well as in areas involved in attention, executive functions, and memory formations [76]. Structural and functional modifications in this network may be the biological substrate of the pervasive effect of meditation practice in everyday life. These findings, taken together with previous ones, are leading to new applications of meditation practice in clinical populations and in disease prevention, especially in at-risk groups such as the elderly. In light of recent findings on the potential effect of meditation on age-related cognitive decline [22, 23], it could be intriguing to understand whether neurobiological changes promoted by meditation practice contribute to forming the so-called “Cognitive Reserve” [81]. Possible applications to a wide range of mental disorders affecting self-regulation and self-awareness, such as mood disorders [82, 83], anxiety disorders [84], and substance abuse [85], have also to be considered. In any case, further investigations comparing both psychological and neural effects of meditation practice are needed before any conclusions can be drawn.

## Figures and Tables

**Table 1:** Functional changes in meditators.

Paper	*N*	Contrast	Experience	Meditation
Allen et al., 2012 [37]	61	AFT, task > passive view	6 weeks	MT
Allen et al., 2012 [37]	61	AFT, negative > neutral	6 weeks	MT
Allen et al., 2012 [37]	61	AFT, task by emotion	6 weeks	MT
Brefczynski-Lewis et al., 2007 [44]	41	EM > NM during meditation	—	Buddhist practitioners
Brefczynski-Lewis et al., 2007 [44]	41	EM > INM during meditation	—	Buddhist practitioners
Brefczynski-Lewis et al., 2007 [44]	41	EM > INM meditation > rest, group by task	—	Buddhist practitioners
Creswell et al., 2007 [86]	27	Neural areas associated with MAAS	—	—
Davanger et al., 2010 [34]	4	ACEM meditation > control task	23 years	ACEM
Ding et al., 2014 [87]	32	IBMT > RT	10 days	IBMT
Engström et al., 2010 [36]	8	Meditate	14 months	ACEM and Kundalini
Engström et al., 2010 [36]	8	Word	14 months	ACEM and Kundalini
Engström et al., 2010 [36]	8	Silent mantra	14 months	ACEM and Kundalini
Farb et al., 2007 [42]	27	Experiential focus, MT > controls	8 weeks	MBSR
Farb et al., 2010 [88]	36	Sadness provocation, MT > controls	8 weeks	MBSR
Farb et al., 2013 [89]	36	Interoception > exteroception, MT > controls	8 weeks	MBSR
Grant et al., 2011 [38]	22	Pain, EM > controls	—	Zen
Grant et al., 2011 [38]	22	Hot > warm, EM	—	Zen
Grant et al., 2011 [38]	22	Pain, EM > controls	—	Zen
Guleria et al., 2013 [47]	14	Meditation > control	5.8 ± 0.9 years	SOHAM
Hasenkamp et al., 2012 [43]	14	AWARE-MW	>1 year	FAM
Hasenkamp et al., 2012 [43]	14	SHIFT > MW	>1 year	FAM
Hasenkamp et al., 2012 [43]	14	FOCUS > MW	>1 year	FAM
Hasenkamp et al., 2012 [43]	14	MW > SHIFT	>1 year	FAM
Hasenkamp et al., 2012 [43]	14	Correlations with practice time, AWARE	>1 year	FAM
Hasenkamp et al., 2012 [43]	14	Correlations with practice time, SHIFT	>1 year	FAM
Hasenkamp et al., 2012 [43]	14	Correlations with practice time, FOCUS	>1 year	FAM
Hölzel et al., 2007 [12]	30	Mindfulness > arithmetic, EM	>2 years	Vipassana
Hölzel et al., 2007 [12]	30	EM > controls	>2 years	Vipassana
Ives-Deliperi et al., 2011 [90]	10	Mindfulness > control in EM	8 weeks	MBSR
Jang et al., 2011 [91]	68	EM > controls	39.88 ± 25.58 months	BWVM
Kilpatrick et al., 2011 [92]	32	Auditory/salience	8 weeks	MBSR
Kilpatrick et al., 2011 [92]	32	Medial visual	8 weeks	MBSR
Kilpatrick et al., 2011 [92]	32	Lateral visual	8 weeks	MBSR
Kilpatrick et al., 2011 [92]	32	Sensorimotor	8 weeks	MBSR
Kilpatrick et al., 2011 [92]	32	Executive control	8 weeks	MBSR
Lee et al., 2012 [93]	44	CPT in FAM	>5 years	FAM/LKM
Lee et al., 2012 [93]	44	EPT-happy in FAM	>5 years	FAM/LKM
Lee et al., 2012 [93]	44	EPT-happy in LKM	>5 years	FAM/LKM
Lee et al., 2012 [93]	44	EPT-sad in FAM	>5 years	FAM/LKM
Lee et al., 2012 [93]	44	EPT-sad in LKM	>5 years	FAM/LKM
Lutz et al., 2008 [14]	28	Meditation > resting states, EM > controls	10000 to 50000 hours	Buddhist practitioners
Lutz et al., 2009 [94]	22	Meditation > resting states, EM > controls	10000 to 50000 hours	Buddhist practitioners
Lutz et al., 2013 [39]	28	Hot > warm, EM > controls	>10000 hours	Buddhist practitioners
Lutz et al., 2013 [39]	28	EM > controls	>10000 hours	Buddhist practitioners
Manna et al., 2010 [56]	8	FAM > rest, in EM	Mean 15750 hours	Buddhist monks
Manna et al., 2010 [56]	8	OM > FAM, in EM	Mean 15750 hours	Buddhist monks
Manna et al., 2010 [56]	8	OM > rest, in EM	Mean 15750 hours	Buddhist monks
Mascaro et al., 2013a [40]	29	Self pain task, pain > no pain	8 weeks	CBCT
Mascaro et al., 2013a [40]	29	Other pain tasks, pain > no pain	8 weeks	CBCT
Mascaro et al., 2013b [41]	29	RME, emotion > gender	8 weeks	CBCT
Monti et al., 2012 [95]	8	Post- > pretreatment	8 weeks	MBAT
Monti et al., 2012 [95]	8	Post- > pretreatment, MBAT > controls	8 weeks	MBAT
Orme-Johnson et al., 2006 [96]	24	Post- > pretreatment	31.3 ± 2.3 years	TMT
Taylor et al., 2011 [45]	22	Positive > neutral pictures	1000 hours	Zen
Tang et al., 2013 [46]	60	IBMT > RT	10 sessions	IBMT
Tang et al., 2013 [46]	60	IBMT, post > pre	10 sessions	IBMT
Wang et al., 2011 [97]	10	Meditation 1 > control	30 years	Kundalini
Wang et al., 2011 [97]	10	Meditation 2 > control	30 years	Kundalini
Wang et al., 2011 [97]	10	Meditation 2 > Meditation 1	30 years	Kundalini
Wang et al., 2011 [97]	10	Baseline 2 > Baseline 1	30 years	Kundalini
Xu et al., 2014 [33]	14	NDM > rest	27 ± 9 years	ACEM
Xu et al., 2014 [33]	14	Concentrative practicing > rest	27 ± 9 years	ACEM
Xu et al., 2014 [33]	14	NDM > concentrative practicing > rest	27 ± 9 years	ACEM

*Notes*. AST: affective Stroop task; MT: mindfulness training; EM: expert meditators; NM: novice meditators; INM: incentive novice meditators; MAAS: Mindful Attention Awareness Scale; IBMT: integrative body-mind training; RT: relaxation training; MBSR: mindfulness-based stress reduction; BWVM: brain-wave vibration meditation; FAM: focused attention meditation; LKM: loving-kindness meditation; CPT: continuous performance test; EPT: emotion-processing task; OM: open monitoring meditation; CBCT: cognitively based compassion training; RME: reading the mind eyes test; MBAT: mindfulness-based art therapy; TMT: transcendental meditation technique; NDM: nondirective meditation; MW: mind wondering.

**Table 2:** sMRI studies on expert meditators.

Paper	*N*	Contrast	Experience	Meditation
Kang et al., 2013 [49]	92	Meditators versus controls	41.23 ± 27.57 months	BWV
Wei et al., 2013 [54]	40	Meditators versus controls	14 ± 8 years	TCC
Hölzel et al., 2011 [48]	16	Pre- to post-MBSR training	8 weeks	MBSR
Kurth et al., 2014 [50]	100	Meditators versus controls	19.8 ± 11.4 years	—
Kurth et al., 2014 [50]	100	Correlation with meditation practice	19.8 ± 11.4 years	—
Leung et al., 2013 [51]	25	Meditators versus controls	>5 years	LKM
Luders et al., 2009 [6, 52]	44	Meditators versus controls	24.18 ± 12.36 years	—
Luders et al., 2009 [6, 52]	44	Meditators versus controls	24.18 ± 12.36 years	—
Luders et al., 2013 [24]	100	Meditators versus controls	19.8 ± 11.4 years	—
Vestergaard-Poulsen et al., 2009 [53]	20	Meditators versus controls	16.5 ± 5.1 years	Tibetan Buddhism

*Notes*. BWV: brain-wave vibration; TCC: Tai Chi Chuan; MBSR: mindfulness-based stress reduction; LKM: loving-kindness meditation.
